# Poliovirus receptor (PVR) expression as a predictor of relapse in colorectal cancer: bioinformatics and virtual screening

**DOI:** 10.3389/ebm.2026.10745

**Published:** 2026-02-13

**Authors:** Sulaiman S. Alhudaithi, Muhamed Hamza R. Salih, Zaid H. AlHusseini, Sarah M. Almufadhili, Noura Alelayani, Ahmed H. Bakheit, Hamad M. Alkahtani, Hanadi H. Asiri, Ali A. Alshamrani, Ali R. Alhoshani, Moureq R. Alotaibi, Homood M. As Sobeai

**Affiliations:** 1 Department of Pharmaceutics, College of Pharmacy, King Saud University, Riyadh, Saudi Arabia; 2 Department of Pharmacology and Toxicology, College of Pharmacy, King Saud University, Riyadh, Saudi Arabia; 3 Department of Pharmaceutical Chemistry, College of Pharmacy, King Saud University, Riyadh, Saudi Arabia

**Keywords:** colorectal cancer, poliovirus receptor, prognostic biomarker, relapse, virtual screening

## Abstract

Colorectal cancer (CRC) is one of the most frequently diagnosed malignancies worldwide. Despite advancements in CRC treatment strategies in recent years, disease recurrence remains a major problem; relapsed patients have a poorer prognosis and higher mortality risk. Several factors have been associated with CRC relapse. However, the role of immune checkpoints in CRC recurrence remains elusive. In this work, we aimed to investigate immune checkpoint genes correlated with recurrence in CRC, evaluate their potential as prognostic biomarkers, and identify promising immune checkpoint inhibitors through molecular docking and molecular dynamics simulations. Clinical, genetic, and epigenetic data of relapsed and relapse-free CRC patients in the Cancer Genome Atlas were retrieved from the cBioportal database and evaluated. Subsequently, molecular docking and molecular dynamics simulations studies were conducted to identify suitable poliovirus receptor (*PVR*)/TIGIT binders. *PVR* is a ligand for TIGIT and competes with CD226. The crystal structure used for docking was obtained from the Protein Data Bank (PDB ID: 3UDW). Using this investigative approach, clinical parameters data revealed that among immune checkpoint genes, the *PVR* gene was significantly upregulated in relapsed patients. That upregulation was strongly correlated with diagnosis age, Aneuploidy, fraction genome alterations, and mutation count. Furthermore, free survival analysis showed that patients exhibiting elevated *PVR* levels were 2.16 times more likely to relapse than those with low *PVR* expression (*p* = 0.039). Virtual screening identified 106 natural compounds as potential binders at the *PVR*/TIGIT interface. Molecular docking and molecular dynamics simulations identified three binders that exhibit favorable interactions with *PVR*, with ZINC001848443492 emerging as the most promising. The results underscore the potential role of *PVR* as a prognostic biomarker for relapse in CRC. Future studies, including TIGIT-PVR blockade assays and assessments of the impact of predicted PVR/TIGIT interface binders on T cell function, are necessary to validate this study’s findings.

## Graphical Abstract

**Figure:**
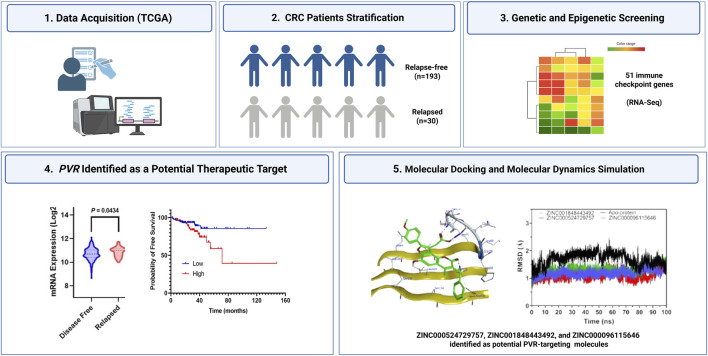

## Impact statement

Poliovirus receptor (PVR) upregulation has been associated with relapse in many malignancies, such as lung and cervical cancer. However, the association between PVR overexpression and colorectal cancer (CRC) recurrence has not been established. This work demonstrates, for the first time, a correlation between PVR gene upregulation and relapse in CRC patients. It also reveals that certain clinical and genetic factors were associated with high PVR levels. Our study also showed that CRC patients overexpressing PVR were more susceptible to recurrence. In addition, three natural compound inhibitors that effectively target PVR were discovered through molecular docking and dynamics simulations, introducing promising therapeutic candidates for preventing relapse. These findings advance the field by unveiling the role of PVR in CRC recurrence and providing a rationale for the preclinical assessment of the identified immune checkpoint inhibitors, thereby opening new avenues for novel treatment strategies that could improve patient outcomes and reduce relapse rates.

## Introduction

Colorectal cancer (CRC) is the third most prevalent malignancy and a leading cause of cancer-related deaths worldwide [1, 2]. The five-year survival rate of CRC in the localized stage is over 85%; however, in the distant stage of CRC, the rate drops dramatically to less than 20% [3]. CRC metastasizes primarily to the liver and, to a lesser extent, to the lungs, brain, and peritoneum [4]. Liver metastasis develops in 20%–50% of CRC patients, significantly deteriorating their prognosis and greatly contributing to the poor survival rates in the advanced stages [5, 6]. Current treatment strategies for CRC are diverse and tailored based on stage, cancer type, and patient genetic profile. Strategies include surgery (for resectable tumors), chemotherapy, targeted therapy, and immunotherapy [4, 7].

The tumor microenvironment (TME) in CRC is complex and besides cancer cells, it includes blood vessels, resident and infiltrating immune cells of myeloid origin such as dendritic cells (DC) and tumor-associated macrophages (TAMs), as well as lymphoid populations like T helper 1 (Th1), T regulatory (Treg), and CD8^+^ cytotoxic T cells, along with natural killer (NK) cells. In addition, the TME encompasses stromal cells and their associated extracellular matrix (ECM) proteins [8]. Solid tumor evasion is mediated, in part, by tumor-induced cytotoxic T cell exhaustion through upregulation of co-inhibitory molecule expression [9]. ICIs have emerged as an efficacious immunotherapeutic strategy for CRC, and they act by targeting immune checkpoint axes such as PD-1-PD-L1/PD-L2 and CTLA4-CD80/CD86, reversing T cell exhaustion, and thereby improving the adaptive antitumor response [10]. Notably, tumors with microsatellite instability (MSI-H)/deficient mismatch repair (dMMR) respond better to PD-1/PD-L1 immunotherapy, compared to tumors with microsatellite stability (MSS) or proficient mismatch repair (pMMR)/low microsatellite-instability (MSI-L) [11]. However, ICIs targeting the PD-1-PD-L1 are effective only in tumors that highly express co-inhibitory molecules like PD-L1 [12, 13]. Further, resistance to those therapies has emerged as a formidable challenge in cancer treatment [14, 15]. Other immune checkpoints, such as LAG3, VISTA, and poliovirus receptor (PVR), have also been investigated as molecular targets for cancer therapy [16–18]. PVR is a pleiotropic protein, highly expressed in certain tumors. It acts as a ligand for T cell immunoreceptor with Ig and immunoreceptor tyrosine-based inhibitory motif, ITIM, domains (TIGIT), CD96, and to a lesser extent, CD226, and upon its interaction with the inhibitory receptors (CD96 and TIGIT), transmits a negative signal to T cell activation [19, 20]. *PVR* upregulation has been associated with tumor progression in lung, pancreatic, and cervical cancers [19, 21]. Moreover, in CRC, *PVR* is highly expressed, and its elevated levels are correlated with poor prognosis [19, 22]. To date, no FDA-approved ICI targets the PVR-TIGIT axis. Therefore, a therapeutic strategy targeting PVR/TIGIT represents an unmet clinical need in CRC, particularly in MSS and pMMR/MSI-L patients. Despite substantial advances in CRC treatment over the recent decades, the survival rates, particularly in advanced stages, remain poor and recurrence rates continue to be a significant concern.

Although many patients undergo curative tumor resection/treatment, the malignancy tends to recur within a few years in some populations [23]. Recurrence occurs in approximately 30% of patients with stage I-III and can reach up to 65% in those with stage IV [23]. Notably, CRC patients who experience recurrence within 5 years following surgery/treatment have a substantially increased risk of mortality [24]. Various clinical and molecular characteristics have been associated with CRC recurrence, including genetic and epigenetic factors, tumor features, and treatment-related factors [25–27]. The most frequently mutated genes in CRC patients are *APC*, *TP53*, *KRAS*, and *PIK3CA* [28], and these genes are also prevalent in CRC patients who developed tumor relapse after undergoing curative surgery. A study by Lan et al. demonstrated that *KRAS* gene is the most frequently mutated in both early and late recurrence of colon cancer, followed by mutations in *TP53*, *PIK3CA*, and *ABC*. In rectal cancer, however, *TP53* mutations were the most common among patients with recurrent CRC [27]. Epigenetic factors, including *CDKN2A* hypermethylation and methylation of *HPP1* and *HLTF* have also been widely linked to an elevated risk of recurrence and tumor progression in CRC patients [29]. Tumor-related factors associated with relapse in CRC include the cancer stage; with more advanced stages, the probability of recurrence increases [23, 24]. The likelihood of relapse is also influenced by the type of CRC malignancy; rectal cancer has higher recurrence rates compared to colon cancer [30].

The study aimed to unveil potential prognostic biomarkers and identify molecular targets that may be suitable for alternative therapeutic approaches in CRC. In this work, we analyzed data from CRC patients to identify novel genes associated with recurrence, with a particular emphasis on immune checkpoint genes. In addition, molecular docking and molecular dynamics simulations were conducted to identify potential binders/hits targeting the immune checkpoint axis of interest.

## Materials and methods

### Study design and data collection

Clinical, genetic, and epigenetic data related to CRC patients (n = 223) of the Cancer Genome Atlas (TCGA) [31] were extracted and analyzed using cBioProtal database (https://www.cbioportal.org/, 14 January 2023) [32–34]. Patients were allocated into two groups based on relapse status: patients who had undergone recurrence (n = 30) and relapse-free subjects (n = 193).

### Clinical characteristics, genetic, and epigenetic factors assessment

Data of clinical characteristics (cancer type, stage, diagnosis age, weight, gender, and vital status), Genetic factors (mutations, aneuploidy, buffa hypoxia, and MSI MANTIS), and epigenetic factors (methylation status) from both relapsed and relapse-free CRC patients were examined statistically using Student’s t-test (continuous variables) and Chi-square/Fisher’s Exact test (Categorical variables). Differences with *P* values <0.05 indicate statistical significance.

### Immune checkpoint genes screening

To screen for upregulated immune checkpoint (ICP) genes among relapsed CRC patients relative to relapse-free patients, RNA-Sequencing (RNA-Seq) data were utilized. The expression of 51 genes including ICPs, human leukocyte antigen (HLA), cell adhesion, and co-stimulatory genes in all patients was assessed. Expression data were presented as normalized mean log_2_ values ±SEM. Log2 ratios of expression values in the replace-free group relative to the relapsed group were computed. Statistical significance between the two cohorts was assessed using Student’s t-test. Differences of *P* value <0.05 were considered statistically significant.

### Genetic alteration and methylation status examinations

To explore the mechanisms behind the upregulation of candidate ICP genes, both genetic factors (somatic mutations and putative copy number alterations [PCNA]) and epigenetic modifications (methylation status) were assessed. Somatic mutations analyzed included missense, inframe, and truncated mutations. PCNA analysis identified amplifications and deletions in the examined ICP genes. The log_2_ ratio was calculated. Statistical significance was assessed using a two-sided Fisher’s Exact test, with a significance threshold of p < 0.05. For methylation status, the average methylation levels of the ICP genes were calculated for each cohort, and the log_2_ ratio was determined. A Student’s t-test was used to evaluate the statistical significance of differences in methylation levels.

### Correlation assessment between clinical parameters and the upregulated ICP gene expression

Clinical parameters, including cancer type, stage, diagnosis age, weight, gender, vital status, mutations count, aneuploidy score, buffa hypoxia score, MSI MANTIS, associated with the upregulated immune checkpoint genes in CRC patients were evaluated. Student’s t-test was utilized to determine statistical significance (p < 0.05) between data from relapsed and relapse-free patients. Pearson correlation coefficient (r) was also used to identify the correlation between the stated factors and the upregulated immune checkpoint genes. The upregulated genes were further analyzed in relation to MSI status (MSI-high vs. MSI-low) to determine whether the observed associations were MSI-dependent.

### Survival studies

To investigate the influence of upregulated immune checkpoint genes on overall and free survival, Kaplan–Meier survival analyses were employed and curves were generated based on RNA-Seq data for individual gene expressions. Patients were stratified into two cohorts based on the upregulated gene expression levels, with the mean expression value serving as the threshold: one group exhibited high gene expression (≥median), while the other showed low expression (<median). In addition, we evaluated the impact of MSI MANTIS score on disease-free survival to determine whether MSI status influences relapse risk. The statistical significance between these cohorts was assessed by calculating hazard ratios (HRs) and corresponding P-values from the Log-rank (Mantel-Cox) test. GraphPad Prism 9.1 was used for data analysis. The association between the candidate ICP genes and disease-free survival was validated in an independent large study, GSE39582, which comprises 566 primary colon tumors profiled by transcriptomic analysis using the Affymetrix Human Genome U133 Plus 2.0 Array [35]. The Kaplan–Meier figures of the validating dataset were generated using KM plotter [36].

### Virtual screening

#### Ligand preparation

The crystal structure of the human TIGIT/PVR complex (PDB ID: 3UDW) [37] was retrieved from the Protein Data Bank (https://www.rcsb.org/). During preprocessing, all non-protein atoms, including water molecules, were excluded from the structure. The three-dimensional conformation of PVR (chain C of the 3UDW structure) was utilized for subsequent computational docking studies.

A dataset comprising 80,617 natural product compounds was obtained at no cost from the ZINC20database (https://zinc.docking.org/substances/subsets/natural-products/). Before screening, the compounds were processed using RDKit tools to remove salts and perform complex structural refinements, ensuring their chemical integrity and accurate representation of their molecular structure.

#### Protein preparation and site finder

For molecular docking studies, the three-dimensional conformation of PVR (chain C) from 3UDW, which represents the complex of human TIGIT bound to the PVR/CD155 D1 domain, was selected. The structure was solved by X-ray diffraction at a resolution of 2.90 Å, providing moderate-quality atomic coordinates suitable for molecular docking and interface analysis. The structure contains chains corresponding to TIGIT (chains A and B) and PVR/CD155 (chains C and D), forming the native receptor–ligand interface. Preprocessing involved the removal of all non-protein atoms, including water molecules, followed by structure optimization using the Molecular Operating Environment (MOE 2024.06) software (Chemical Computing Group ULC, Montreal, QC, Canada).

The TGIT/PVR complex structure used for docking was obtained from the Protein Data Bank (PDB ID: 3UDW) and imported into MOE for preprocessing. Initial inspection of the structural integrity and stereochemistry was performed using the Structure Preparation tool (MOE | Compute | Prepare | Structure Preparation). The identified issues included incomplete termini, protonation inconsistencies, partial charge warnings, and minor steric clashes, as summarized in the MOE diagnostics (e.g., Termini = 3, HCount = 1, Charge = 1, Protonate3D = 112, Clash = 1). To resolve these issues, the Correct function within the Structure Preparation panel was applied to automatically address missing atoms, standardize residue names, repair incomplete termini, and resolve alternate locations.

Following structural correction, the Protonate 3D tool (MOE | Compute | Prepare | Protonate 3D) was used to assign the optimal protonation states of all ionizable residues at physiological pH, in accordance with local hydrogen-bonding patterns and electrostatic environments. Hydrogens were added, and the hydrogen-bonding network was optimized. Partial charges were then assigned using MOE’s Partial Charges module to ensure proper electrostatic representation for subsequent docking and molecular mechanics refinement.

To relieve local steric clashes and minimize crystallographic strain while maintaining the overall native conformation, a tethered energy minimization step was performed. Minimization was carried out using the Energy Minimize panel with positional restraints applied to backbone heavy atoms to prevent major deviations from the crystal structure. This procedure corrected minor geometric distortions, resolved the detected steric clash, and yielded an energetically optimized receptor conformation suitable for docking. No missing loops were reported for 3UDW; therefore, loop reconstruction was not required. The binding site was identified by selecting residues within a 4.5 Å radius, guided by reference data, and the binding pocket for small molecule binders was determined using the Site Finder module in MOE.

#### Active sites in PVR

The identified β-sheets (Arg68–Leu64 and Phe125–Tyr121) and loop region (His69–Met75) in the PVR receptor represent critical structural elements involved in its interaction with the TIGIT protein. These regions form part of the binding interface and are positioned at the surface of the immunoglobulin-like domain, enabling direct contact with TIGIT. Therefore, the active sites of PVR responsible for binding are located within these specific loop and sheet regions. The TIGIT-PVR interface is formed through a highly complementary lock-and-key arrangement involving residues located primarily on the C′C″ loop and FG loop of the IgV domains of both molecules. The AX_6_G motif (residues 76–83 in PVR and 66–74 in TIGIT), located on the C′C″ loop, forms a conserved hydrophobic pocket that functions as the “lock.” This concave pocket is capped by the terminal residue of the motif and provides a structurally rigid anchoring site. Opposing this, the FG loop contributes the corresponding “key” element, defined by the T(F/Y)P motif—residues 127–129 in PVR and 112–114 in TIGIT—where an aromatic residue (F128 in PVR or Y113 in TIGIT) inserts directly into the hydrophobic lock pocket of the partner molecule. These complementary topologies on symmetric corners of the interface constitute the core of the TIGIT-PVR binding specificity.

Additional stabilizing contacts arise from the conserved (V/I)(S/T)Q motif—residues 61–63 in PVR and 54–56 in TIGIT—which further supports intermolecular packing across the β-sheet interface (A′GFCC′C″ region). Collectively, these residues bury approximately 1,600 Å^2^ of surface area and define the dominant hotspots governing the TIGIT/PVR interaction. Notably, the same residues form the binding surface exploited by poliovirus, highlighting the functional significance and evolutionary conservation of this interaction site [37, 38].

#### Molecular docking of small molecules targeting PVR

The crystal structure of the TIGIT-PVR complex (PDB ID: 3UDW) was utilized as a structural basis for identifying small molecules capable of effectively disrupting TIGIT-PVR interactions. Key residues located at the interaction interface between TIGIT and PVR were recognized as critical for mediating their binding, and this region was chosen as the primary target for molecular docking. Ligand placement was performed using the Triangle Matcher method, and the initial poses were evaluated using the London dG scoring function, which estimates the binding free energy based on empirical terms and hydrophobic contact potentials. For each ligand, 30 preliminary poses were generated and ranked according to their London dG scores. The top poses were subsequently subjected to refinement using the Rigid Receptor protocol, during which the GBVI/WSA dG scoring function was applied to rescore and estimate the binding affinity. GBVI/WSA dG combines the Generalized Born Volume Integral implicit solvation model with Weighted Surface Area terms to provide a more accurate approximation of binding free energy during refinement. From this stage, 10 refined poses were retained. Together, the London dG (primary scoring) and GBVI/WSA dG (refinement scoring) functions provided a consensus evaluation of ligand binding, ensuring both rapid screening and more physically grounded energy estimation. As detailed in the screening protocol, the PVR domain underwent structural refinement, 3D protonation, and optimization. Additionally, energy minimization was applied to 80,617 natural product compounds from the ZINC database (https://zinc.docking.org/substances/subsets/natural-products) using MOE software. From those, 29,308 molecules that complied with Lipinski’s rule of five and had molecular weights between 250 and 550 Da were selected. Based on predicted binding modes, 166 compounds were identified to interact specifically with the PVR interface. Subsequent molecular docking was performed, and compounds showing docking scores (S-values) of ≤−10 were prioritized for further consideration.

#### Molecular dynamics simulation studies

Molecular dynamics (MD) simulations were conducted using Nanoscale Molecular Dynamics (NAMD) software (version 3.0) [39]. These simulations involved both the PVR protein alone and its complexes with the ligands ZINC000096115646, ZINC001848443492, and ZINC000524729757. Initial energy minimization of each ligand–PVR complex was carried out using the MOE software suite (version 2024.10). Complex configuration files were created through CHARMM-GUI [40], and the systems were parameterized using the CHARMM General Force Field (CGenFF) [41, 42]. The all-atom additive CHARMM36 force field was applied to construct the topologies of the ligand–PVR complexes. Solvation was performed using the TIP3P water model [43]. Following this, the system—comprising the complexes, ions, and solvent—underwent energy equilibration and a minimization step of 10,000 iterations. The MD simulation was then run for 1,000,000 steps, including a production phase of 125,000 steps. The equilibration period was set to 250 ps using the NVT ensemble, whereas production runs were carried out under the NPT ensemble for 100 ns. Finally, the stability of both the unbound PVR and its complexes was evaluated using VMD software [44] through the analysis of root-mean-square deviation (RMSD), root-mean-square fluctuation (RMSF), and radius of gyration (Rg) [45, 46].

## Results

### Clinical characteristics and genetic features of the enrolled patients

Patients’ clinical data and their genetic profiles were collected and allocated into two groups based on relapse status: i) Relapse-free and ii) relapsed. Results (Table 1) showed that CRC type, stage, patient’s age at diagnosis, and weight, were not associated with recurrence status. Interestingly, nearly twice as many male CRC patients experienced recurrence compared to females, while no gender differences were observed in relapse-free subjects. However, such differences were not statistically significant (*p* = 0.09). Vital status, on the other hand, was markedly correlated with relapse; the mortality rate in the relapsed group was significantly higher compared to the relapse-free cohort (*p* < 0.0001).

**TABLE 1:** Clinical characteristics and genetic factors of relapse-free and relapsed CRC patients.

Factor	Relapse-free n = 193 (86.55%)	Relapsed n = 30 (13.45%)	*P* value ​
Cancer type	Rectal adenocarcinoma = 36 (69.43%) Colon adenocarcinoma = 134 (18.65%) Mucinous adenocarcinoma of the colon and Rectum = 23 (11.92%)	Rectal adenocarcinoma = 6 (76.67%) Colon adenocarcinoma = 23 (20.00%) Mucinous adenocarcinoma of the colon and rectum = 1 (3.33%)	0.369
Stage	I = 48 (24.87%) II = 81 (41.97%) III = 57 (29.53%) IV = 0 (0.00%) Unknown = 7 (3.63%)	I = 4 (13.33%) II = 15 (50.00%) III = 10 33.33%) IV = 0 (0.00%) Unknown = 1 (3.33%)	0.370​
Diagnosis age (years)	64.97 ± 0.88	65.57 ± 2.47	0.809​
Weight (kgs)	78.43 ± 2.23	82.03 ± 4.23	0.371​
Gender	Female = 96 (49.74%) Male = 97 (50.26)	Female = 10 (33.33%) Male = 20 (66.67%)	0.094​
Vital status	**Living = 184 (95.34%)** **Deceased = 9 (4.66%)**	**Living = 21 (70.00%)** **Deceased = 9 (30.00%)**	**<0.0001**​
MSI MANTIS score	0.42 ± 0.02	0.39 ± 0.04	0.050
Mutation count	Positive = 5 (4.59%) Negative = 91 (83.49%) Unknown = 13 (11.93%)	Positive = 7 (7.86%) Negative = 67 (75.28%) Unknown = 15 (16.85%)	0.356
Aneuploidy score	11.35 ± 0.58	14.43 ± 1.56	0.055
Buffa hypoxia score	18.77 ± 1.34	19.29 ± 3.34	0.879
Fraction genome alteration	0.23 ± 0.01	0.27 ± 0.03	0.225

Values are mean ± SE or frequencies, as appropriate. P < 0.05 was considered statistically significant. Significant values are in bold.

In addition, genetic data indicated that the average aneuploidy score was marginally higher in the relapsed cohort, whereas the mean MSI MANTIS score was slightly lower. No significant differences in the other genetic factors were observed between the two study groups (Table 1). Details of the clinical characteristics and the genetic factors are listed in Supplementary Table S1.

### Immune checkpoints and other cell surface proteins gene expression in relapsed and relapse-free subjects

**FIGURE 1:**
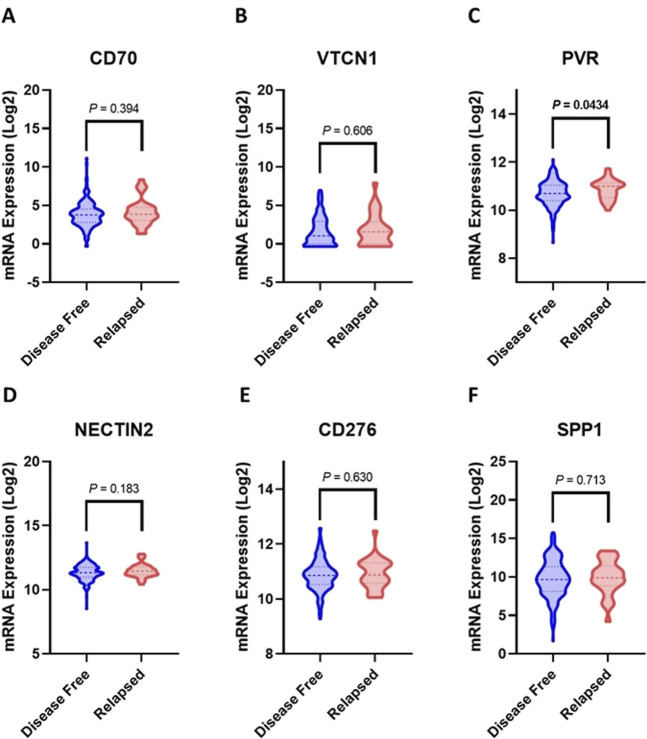
Upregulated Immune checkpoint genes in relapsed CRC patients relative to disease-free. **(A)** *CD70*, **(B)** *VTCN1*, **(C)** *PVR*, **(D)** *NECTIN2*, **(E)** *CD276*, **(F)** *SPP1*. The mean and 95% confidence interval values are represented by bold and light intermittent lines, respectively. Statistical significance was determined by Student’s t-test.

RNA-Seq data of the enrolled CRC patients were retrieved and analyzed to screen for ICIs associated with disease recurrence. Alterations in other genes, including HLA, cell adhesion, and co-stimulatory genes, were also investigated. Among the examined ICIs, *PVR* was significantly correlated with CRC recurrence (Figure 1C). *NECTIN2* (also known as Poliovirus receptor-related 2, PVRL2) expression is slightly elevated in patients who experienced recurrence compared to the relapse-free group (*p* < 0.2) (Figure 1D). In contrast, the expression of *CD274* (PD-L1) and *PDCD1* (PD-1) genes was relatively higher in relapse-free subjects (Supplementary Table S2). Nevertheless, except for *PVR*, no significant differences in immune checkpoint gene profiles were detected between the relapsed and the relapse-free group (Figure 1; Supplementary Table S2).

Antigenic peptides, including cancer antigens, are presented to CD4 + T cells in the context of HLA class II molecules, such mechanism is crucial for T cell activation and function [47]. CD4 + T helper 1 cells promote cytotoxic T cell differentiation and proliferation, thereby enhancing the adaptive antitumor response [47, 48]. RNA-Seq datasets revealed that *HLA-DRA* (a type of HLA class II molecules) gene expression was significantly lower in the relapsed CRC group relative to the relapse-free group (Supplementary Figure S1A). Other *HLA* class II types, including HLA-DRB1, HLA-DMA, HLA-DMB, HLA-DQA1, and HLA-DPA1, were also downregulated in the relapsed cohort relative to the relapse-free group; however, such differences did not reach statistical significance (*p* = 0.08–0.15) (Supplementary Figure S1). These data suggest that the antigen presentation capacity is reduced in the relapsed CRC patients compared to relapse-free subjects, which in turn may compromise the antitumorigenic functions of T cells.

### Genetic alteration and methylation status of identified upregulated ICP genes

The genetic alteration, including somatic mutation and PCNA, and methylation status in the upregulated ICP genes, *PVR*, were examined. No significant somatic mutations or abnormal PCNA were detected in *PVR* in the relapsed group relative to the relapse-free group (Supplementary Table S3). In addition, hypermethylation of *PVR* was observed in the relapsed group compared to the disease-free cohort; however, the comparison did not result in statistical significance (*p* = 0.127, Supplementary Table S4). It is worth mentioning that *APC (70.2%), TP53 (55.1%), TNN (49.5%), KRAS (41.9%), and PIK3CA (31.8%)*, were the most frequently mutated genes in our cohort, with no significant difference between the relapse-free and relapsed groups (Supplementary Table S5).

### Correlation between clinical parameters and the upregulated immune checkpoint gene expression

The potential correlation between *PVR* gene expression and CRC patients’ clinical and genetic factors was assessed. Amongst all the evaluated clinical factors, the patient’s age at diagnosis was the only factor strongly correlated with elevated *PVR* gene expression (Table 2), *PVR* upregulation was more common in younger patients than in older subjects. Genetic data, on the other hand, revealed several factors associated with upregulated *PVR* levels (Table 2). Aneuploidy and fraction genome alterations were positively correlated with *PVR* upregulation (*p* = 0.017 and *p* = 0.005, respectively), whereas mutation count was negatively correlated (*p* = 0.04). These data indicate that *PVR* elevation is somewhat associated with the “nature” of genetic alteration rather than the “number” of mutations. MSI status (high vs. low) did not significantly influence *PVR* expression (Supplementary Figure S2A). No significant correlations were detected between *PVR* increased gene expression and any of the other evaluated factors, including cancer type, stage, weight, gender, vital status, and buffa hypoxia score (Table 2).

**TABLE 2:** Clinical and genetic factors and their correlation with PVR upregulation.

Factor	Pearson correlation coefficient (r)	*P* value ​
Cancer type	-	0.063
Stage	-	0.083
Diagnosis age	**−0.133**	**0.049**
Weight	0.107	0.267
Gender	-	0.636
Vital status	-	0.99
Mutation count	**−0.147**	**0.040**
Aneuploidy score	**0.161**	**0.017**
Buffa hypoxia score	0.084	0.349
Fraction genome alteration	**0.191**	**0.005**
MSI MANTIS score	−0.126	0.071

P < 0.05 was considered statistically significant using Pearson correlation coefficient. Significant values are in bold.

### Survival analysis

**FIGURE 2:**
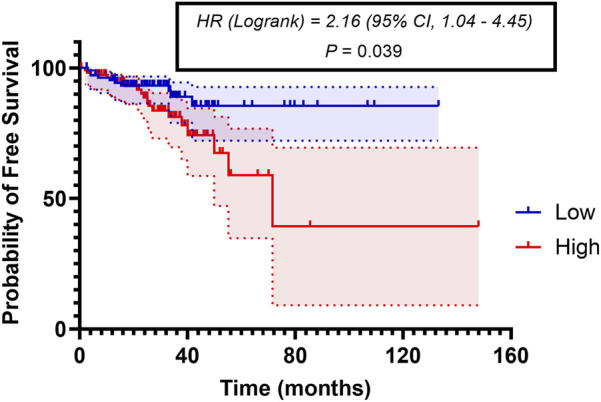
Free survival curves for patients with low *PVR* expression (blue) and patients with high *PVR* expression (red). The median gene expression value was used as the cut-off to stratify patients into the two cohorts, high and low. Hazard ratio (HR) and *p*-value were calculated using the log-rank test. The shaded area around each curve represents 95% confidence interval.

Considering the correlation between *PVR* gene overexpression and recurrence identified in this study, and the established association between relapse and survival outcomes observed both here and in previous work [24], we were prompted to further elucidate the association between *PVR* and relapse in CRC and also explore the relationship between *PVR* gene expression and survival outcomes. Thus, both overall and free survival analyses were performed and the survival data from the high-*PVR* patients’ group were compared with low-*PVR* patients’ group data. Kaplan–Meier analysis showed that overall survival was not significantly impacted by *PVR* genetic levels in CRC patients (Supplementary Figure S3). However, disease-free survival investigation showed that patients with tumors expressing high *PVR* had higher relapse rates (Hazard ratio: 2.016 *P* = 0.039) (Figure 2). These results were validated in the GSE39582 cohort (Supplementary Figure S4). *PVR* expression was significantly associated with disease-free survival, with patients exhibiting high *PVR* expression showing a higher risk of relapse compared with those with low *PVR* expression (HR = 1.46, 95% CI: 1.08–1.97; p = 0.013). These results independently support our findings and further substantiate the prognostic relevance of *PVR* for relapse risk. In addition, we conducted survival analyses stratified by MSI status. Patients were classified as MSI-high or MSI-low, and no significant difference in disease-free survival was observed between the two groups (Supplementary Figure S2B).

### Docking of compounds 1–166 with PVR

**FIGURE 3:**
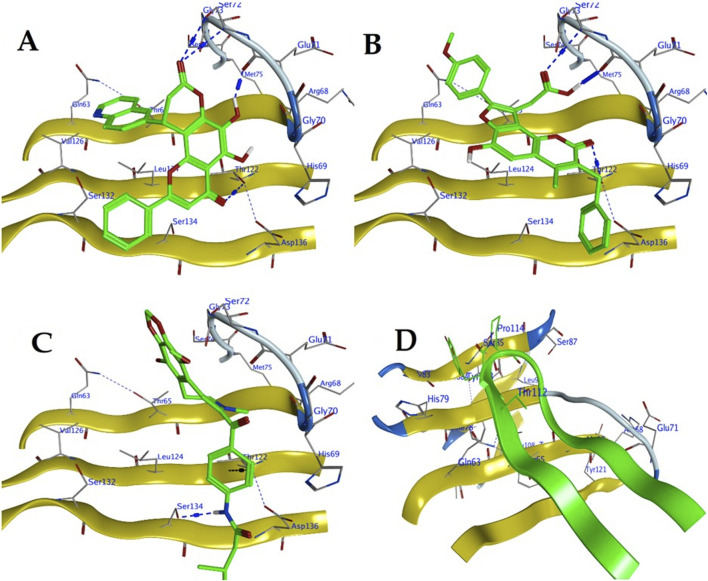
Molecular docking representations of the active site of the poliovirus receptor (PVR/CD155) interacting with **(A)** ZINC000096115646, **(B)** ZINC001848443492, **(C)** ZINC000524729757, and **(D)** TIGIT, highlighting key binding residues and interactions. The yellow ribbons depict the PVR structure, while green structures represent the ligands or TIGIT, with specific amino acid residues (e.g., Ser132, Glu70, His187) and hydrogen bonds (blue dashed lines) illustrating the binding interfaces.

The molecular docking analysis identified three natural product ligands—ZINC000096115646, ZINC001848443492, and ZINC000524729757—that display favourable binding interactions with the PVR receptor at its TIGIT-binding interface (Figure 3).

ZINC000096115646 forms multiple hydrogen bonds with residues GLU71, SER72, and THR122 in chain C of PVR, contributing to a cumulative binding energy (E) of −1.6 kcal/mol and an S-value of −11.02 kcal/mol. Notably, its hydrogen bond with GLU71 (2.82 Å) and a π-H interaction with LEU124 (4.02 Å) suggest a stable and well-oriented binding pose within the active site region (Figure 3A; Table 3).

**TABLE 3:** The molecular docking interactions between selected natural product ligands and the PVR receptor at the TIGIT-binding interface.

Compound	Ligand	Receptor	Interaction	Distance	E (kcal/mol)	S (kcal/mol)
ZINC000096115646	O 27	O GLU 71 (C)	H-donor	2.82	−1.6	−11.02
	O 1	CA SER 72 (C)	H-acceptor	3.51	−0.8	
	O 1	OG SER 72 (C)	H-acceptor	2.99	−1.1	
	O 34	OG1 THR 122 (C)	H-acceptor	3.33	−0.8	
	6-ring	CD2 LEU 124 (C)	pi-H	4.02	−1.2	
ZINC001848443492	O 52	O GLU 71 (C)	H-donor	2.73	−6.5	−10.56
	O 42	OG1 THR 122 (C)	H-acceptor	3.23	−0.6	
	O 51	CA SER 72 (C)	H-acceptor	3.53	−0.9	
ZINC000524729757	N 38	OG SER 134 (C)	H-donor	3.1	−1.4	−10.26
	6-ring	OG1 THR 122 (C)	pi-H	3.47	−0.9	

ZINC001848443492 also engages key residues at the PVR–TIGIT interface, including GLU71, THR122, and SER72. A strong hydrogen donor interaction with GLU71 (2.73 Å) is associated with a significant interaction energy of −6.5 kcal/mol, which likely contributes substantially to its overall docking score of −10.56 kcal/mol (Figure 3B; Table 3).

ZINC000524729757 forms a hydrogen bond with SER134 and a π-H interaction with THR122. Although the individual interaction energies are modest (−1.4 and −0.9 kcal/mol, respectively), the overall binding remains favorable with an S-value of −10.26 kcal/mol. These interactions indicate a stable association near the receptor’s binding interface (Figure 3C; Table 3).

### Molecular dynamics simulation

**FIGURE 4:**
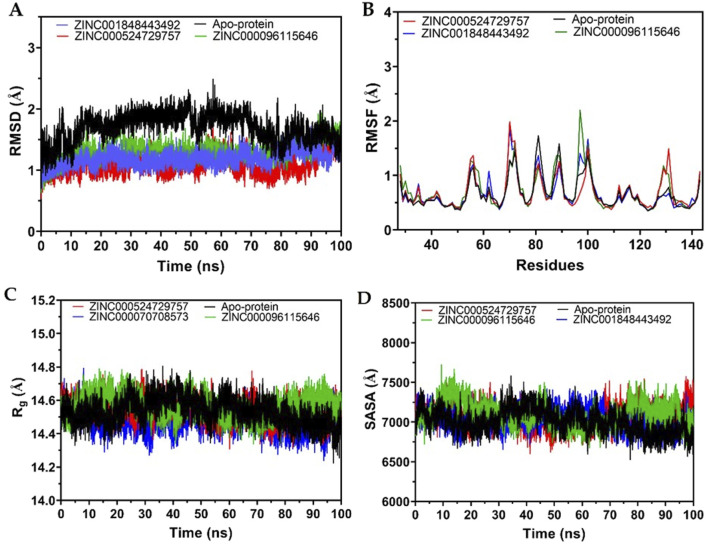
Molecular Dynamics simulation analyses of the poliovirus receptor (PVR/CD155) over 100 ns. **(A)** RMSD (Root Mean Square Deviation) plots comparing the structural stability of PVR in its apo form (black) and when bound to ZINC000096115646 (blue), ZINC001848443492 (red), and ZINC000524729757 (green). **(B)** RMSF (Root Mean Square Fluctuation) plots illustrating per-residue flexibility for the apo-protein (black) and complexes with ZINC000524729757 (red), ZINC001848443492 (blue), and ZINC000096115646 (green). **(C)** Radius of gyration (Rg) plots assessing compactness for the apo-protein (black) and complexes with ZINC000524729757 (red), ZINC000070708573 (blue), and ZINC000096115646 (green). **(D)** SASA (Solvent Accessible Surface Area) plots indicating solvent exposure for the apo-protein (black) and complexes with ZINC000524729757 (red), ZINC000096115646 (green), and ZINC001848443492 (blue).

The Molecular Dynamics (MD) simulation results, as depicted in the provided Root Mean Square Deviation (RMSD) plot over a 100 ns timeframe (Figure 4A), compare the structural stability of the poliovirus receptor (PVR/CD155) in its apo form with its complexes bound to three compounds: ZINC000096115646, ZINC001848443492, and ZINC000524729757. The RMSD values, with means of 1.58 Å for the apo-protein, 1.50 Å for ZINC000096115646, 1.32 Å for ZINC001848443492, and 1.20 Å for ZINC000524729757, indicate that ligand binding generally stabilizes PVR, with ZINC000524729757 showing the lowest mean RMSD, suggesting the strongest stabilizing effect. The standard deviations (0.137 Å for apo-protein, 0.149 Å for ZINC000096115646, 0.117 Å for ZINC001848443492, and 0.178 Å for ZINC000524729757) reveal varying dynamic behaviors, with ZINC001848443492 exhibiting the most consistent structure (lowest STD) and ZINC000524729757 showing the greatest fluctuations, potentially due to a dynamic or less rigid binding mode.

RMSF plot in Figure 4B illustrates the flexibility of the poliovirus receptor (PVR/CD155) residues over a 100 ns MD simulation, comparing the apo-protein form with complexes bound to ZINC000524729757 (red), ZINC001848443492 (blue), and ZINC000096115646 (green). The RMSF values, which indicate per-residue fluctuations, show that the apo-protein (black) exhibits a baseline level of flexibility, while the ligand-bound states display varying degrees of stabilization or increased motion depending on the compound. ZINC000524729757 shows the highest peaks (reaching ∼2 Å), suggesting significant local flexibility, possibly due to dynamic interactions or partial destabilization of certain regions, whereas ZINC001848443492 and ZINC000096115646 exhibit more moderate fluctuations (peaking around 1–1.5 Å), indicating better constraint of PVR’s structure.

The MD simulation results for the radius of gyration (Rg) in Figure 4C and Solvent Accessible Surface Area (SASA) in Figure 4D over a 100 ns timeframe provide insights into the compactness and surface exposure of the poliovirus receptor (PVR/CD155) in its apo form and when bound to ZINC000524729757, ZINC001848443492, and ZINC000096115646. The Rg values, which measure the protein’s compactness, show mean values ranging from 14.482 Å (ZINC001848443492) to 14.566 Å (ZINC000096115646), with the apo-protein at 14.534 Å, indicating that ligand binding slightly alters PVR’s compactness, with ZINC001848443492 promoting the most compact structure and ZINC000096115646 the least. The low standard deviations (0.062–0.077 Å) and narrow ranges between maximum (14.785–14.804 Å) and minimum (14.225–14.334 Å) Rg values suggest stable compactness across all systems, with minimal fluctuations, implying that these compounds do not significantly disrupt PVR’s overall fold, though ZINC001848443492 appears to enhance compactness slightly more effectively.

The SASA results in Figure 4D complement this analysis, with mean values ranging from 7,006 Å^2^ (apo-protein) to 7,104 Å^2^ (ZINC000096115646), indicating that ligand binding generally increases surface exposure, with ZINC000096115646 showing the highest mean SASA, suggesting greater solvent accessibility possibly due to conformational changes or less tight binding. The standard deviations (122.6–151.5 Å^2^) and ranges between maximum (7,419–7,722 Å^2^) and minimum (6,529–6,675 Å^2^) SASA values reflect moderate variability, with ZINC001848443492 exhibiting the lowest STD (122.6 Å^2^), indicating more consistent surface exposure.

## Discussion

CRC is one of the top-ranked malignancies in terms of diagnosis and mortality worldwide [49, 50]. Surgical resection of tumor cells with subsequent adjuvant chemotherapy has long been the gold standard protocol to eliminate CRC [51, 52]. However, disease recurrence plays a pivotal role in increased mortality rates and reduced overall survival in CRC patients [30]. Latest estimates indicate that more than 30% of patients with stage II or III CRC experience recurrence [53, 54]. To date, according to the American Society of Clinical Oncology (ASCO) panel, the accurate definition of ‘high-risk’ relapsed patients remains elusive, as some stage II diagnosed patients and supposedly at higher risk do not experience relapse, whereas some patients with average risk do [55, 56]. Therefore, there is an urgent need to explore reliable predictive molecular biomarkers to accurately identify CRC patients with a higher risk of relapse to help guide treatment decisions.

Over the years, an accumulating number of studies have demonstrated the unprecedented ability of cancer cells to evade destruction by immune cells, which was recently recognized as an independent hallmark of cancer [57]. Tumor cells can suppress immune response signaling in the TME by either downregulating the activity of stimulatory immunoreceptors or upregulating the activity of inhibitory immunoreceptors, also known as “immune checkpoints” [13]. These molecules are expressed on various immune and cancer cells and serve as gatekeepers to prevent overactivation of the immune system [58]. Elevated levels of immune checkpoint genes have been reported in CRC patients and were associated with poorer clinical outcomes [59–61]. However, the role of these checkpoints in CRC therapy failure and disease relapse remains elusive. Interestingly, a recent study showed that PLCG2 is associated with immune evasion and disease progression in CRC and that PLCG2 knockdown enhanced the efficacy of ICI therapy [62]. In this study, we sought to identify immune checkpoint gene-expression signatures at diagnosis and evaluate their utilization as prognostic biomarkers and potential predictors of CRC relapse. We further investigated the genetic and epigenetic mechanisms underlying the differential expression of immune checkpoint genes in relapsed CRC patients compared with their relapse-free counterparts. Bioinformatic analysis of the RNA-sequencing dataset revealed that the immune checkpoint gene, *PVR*, is significantly upregulated in CRC-relapsed patients relative to relapse-free patients. These results suggest that high *PVR* gene expression might have contributed to the intrinsic resistance that the relapsed cohort exhibited, which may have led to the failure of therapy. Furthermore, our data identified several clinical parameters to be significantly associated with elevated levels of *PVR* in CRC-relapsed patients, including diagnosis age, aneuploidy score, and fraction genome alteration. These parameters might be prognostic factors in identifying patients who might have elevated *PVR* expression and a higher risk of relapse.

PVR is a molecule predominantly expressed on myeloid cells and on some cancer cells [63]. Several lines of evidence reported the overexpression of *PVR* in numerous carcinomas, including CRC [22, 64–66]. Higher levels of *PVR* have been strongly associated with disease recurrence in several malignancies, including hepatocellular carcinoma [67], squamous cell lung carcinoma [68], cervical adenocarcinoma [69], and soft tissue sarcomas [70]. In accordance with those studies, our study is the first to demonstrate a significant correlation between elevated expression of the *PVR* gene and the incidence of CRC relapse. Similar to the previously published studies [19], we report that higher expression of *PVR* was strongly associated with shorter free survival times compared with the low-expression cohort. These findings have been validated in another large CRC cohort [35], which independently confirmed the prognostic relevance of *PVR* for relapse risk. Furthermore, *PVR* expression was examined according to the MSI MANTIS score (MSI-high vs. MSI-low). We observed no statistically significant difference in *PVR* expression between the MSI-high and MSI-low groups, suggesting that *PVR* expression is MSI-independent. These findings reveal the essential role of PVR in immune-mediated disease relapse.

We analyzed the genetic and epigenetic anomalies of the relapsed patients compared to the relapse-free patients to understand the potential mechanism by which *PVR* was upregulated in the relapsed cohort. No significant mutations or PCNA alterations were found in the relapsed cohort relative to relapse-free cohorts. Although *PVR* was hypermethylated in the recurrence group compared to the non-recurrence group, it failed to reach statistical significance. These data indicate that the upregulation in *PVR* is driven by the mechanisms governing *PVR* gene expression rather than genetic abnormalities. Future mechanistic studies investigating the regulatory factors of *PVR* gene activity in relapsed CRC patients are highly warranted.

Over the last decade, the therapeutic approach of targeting immune-regulating proteins, also known as immunotherapy, to treat solid and non-solid cancers has grown exponentially [71–73]. Of the 11 ICIs approved by the US Food and Drug Administration (FDA), only two have gained accelerated approval to treat patients with metastatic DNA dMMR or MSI-H CRC. Nivolumab (Anti-PD-1) monotherapy or in combination with ipilimumab (anti-CLTA-4) in CRC adult and pediatric patients >12 years old, whose disease has progressed following treatment with at least one agent, including fluoropyrimidine, oxaliplatin, and irinotecan, have shown improved overall response rate (ORR) and duration of response (DOR) [74, 75]. In addition, several ongoing clinical trials are evaluating the safety and efficacy of many ICIs, including Sintilimab and Dostarlimab for the treatment of various types of CRC [76]. Evidence suggests that existing ICIs are poorly effective in the pMMR or MSS CRC population [77]. The landscape of immunotherapy in malignancy is rapidly evolving. Thus, future studies are warranted to explore new targets for treating CRC patients with diverse genetic backgrounds. Since MSS/pMMR CRC largely remains resistant to PD-1/PD-L1 and CTLA-4-based ICIs, and because the TIGIT-PVR axis suppresses CD226 co-stimulation, pharmacologic targeting of PVR, especially when combined with those ICIs, may help convert ICI-refractory pMMR/MSS disease into a responsive state. To our knowledge, our study is the first to show a correlation between PVR upregulation and the development of relapse in CRC patients, and the first to establish the need for further studies to investigate the impact of concomitant ICI use on preventing CRC relapse after complete remission.

Our work was extended to identify natural product ligands targeting PVR by using molecular docking. All three identified ligands demonstrated strong binding affinity, as indicated by their docking scores (S-values), which were ≤−10 kcal/mol, a commonly accepted threshold for significant interaction.

RMSD data suggest that the compounds influence PVR’s conformational dynamics differently, with ZINC000524729757 providing the most significant stabilization despite higher variability, possibly indicating a strong yet flexible interaction, while ZINC001848443492 offers a more rigid and stable binding pose. The apo-protein’s higher mean RMSD (1.58 Å) reflects its intrinsic flexibility without ligand constraints, with an initial adjustment period (0–20 ns) before stabilization (Figure 4A). The reduced RMSD in ligand-bound states highlights the role of these compounds in influencing the active site of PVR. This modulation could affect its natural interactions, such as with TIGIT. However, a more detailed analysis of the binding residues or energetics would be necessary to gain a better understanding of these molecular interactions.

Observations from RMSF data suggest that while all compounds influence PVR’s dynamics, ZINC000524729757 may induce greater conformational variability, potentially reflecting a less rigid binding mode, whereas ZINC001848443492 and ZINC000096115646 provide more consistent stabilization, aligning with their roles in modulating PVR’s active site interactions.

The SASA results suggest that while all compounds influence PVR’s surface properties, ZINC001848443492 maintains a more stable and compact interaction, potentially correlating with its observed lower Rg, whereas ZINC000096115646 may induce a more exposed and less compact conformation, impacting PVR’s interaction dynamics at the active site.

Validating our findings should include binding assays such as surface plasmon resonance (SPR) to assess the binding of our candidate hits to TGIT-PVR, as well as fluorescence-based binding blockade assays to evaluate the efficacy of the candidate compounds in blocking TGIT-PVR interaction [78, 79]. Future research should also explore the impact of TGIT-PVR blockade on CD8^+^ T cell activation, NK cell cytotoxicity, immune cell infiltration, cytokine expression, and tumor burden and recurrence using CRC murine models. The clinical implications of our findings should be cautiously interpreted, given the few limitations of our study. For instance, the reported *PVR* expressions in our study are relative between the CRC-relapsed patients and relapse-free ones. We cannot extrapolate these observations to other subgroups, including early- and late-relapsing patients. Furthermore, we had no control over the inclusion and exclusion criteria of this study’s participating subjects, given that the first part of this study is a secondary analysis of published data sets. The type and length of the initiated chemotherapy, concurrent use of other medications, patient genetic background, time of relapse after a complete remission, and the definition of complete remission are confounders that could have impacted the observed difference between the two cohorts. The fact that this study proposes the concurrent use of ICIs with chemotherapy in PVR-expressing CRC patients as a prophylactic measure to prevent disease relapse is a critical strength of this work.

Our study identifies PVR as a potential drug target and serves as a sturdy foundation for future studies to answer several unresolved questions. In addition to the importance of validating our findings presented in this study, the impact of chemotherapy on the PVR levels, the association between highly-expressed PVR with the timing of relapse after a complete remission, and the role of PVR expression levels with the mortality rates remain open questions that are warranted to be answered.

## Conclusion

In this work, a correlation between the immune checkpoint *PVR* and relapse in CRC was established. RNA-Seq dataset showed that *PVR* is significantly upregulated in relapsed patients compared to relapse-free individuals. Such elevated *PVR* gene levels in patients experiencing recurrence were accompanied by relatively lower levels in genes involved in antigen presentation, and this was specifically observed in HLA-DRA. Further investigation to explore the clinical and genetic factors associated with increased *PVR* gene expression in relapsed CRC patients revealed that diagnosis age, Aneuploidy, fraction genome alterations, and mutation count were strongly correlated with *PVR* upregulation. Furthermore, free survival analysis indicated that patients with tumors highly expressing *PVR* were more susceptible to recurrence compared to *PVR*-low expression patients, verifying the initial screening via RNA-seq analysis. All three natural product PVR/TIGIT interface binders exhibited strong binding affinity, with docking scores (S-values) of ≤−10 kcal/mol and stable interactions with critical residues, including Glu71, Thr122, and Ser72. Among them, ZINC001848443492 emerged as the most promising candidate due to its balanced performance, showing a strong hydrogen bond with GLU71, a docking score of −10.56 kcal/mol, and the most consistent structural behavior during molecular dynamics simulations—evidenced by the lowest RMSD, minimal fluctuation (RMSF), and enhanced compactness and surface stability. Taken together, these findings suggest that *PVR* may work as a prognostic biomarker for recurrence risk in CRC, and that ZINC001848443492 holds potential as a lead compound for the further development of TIGIT-PVR immune checkpoint inhibitors for CRC treatment.

## Data Availability

The datasets analyzed in this study are publicly available at the cBioportal database (Available online: https://www.cbioportal.org/study/summary?id=coadread_tcga_pan_can_atlas_2018) (accessed on 14 January 2023). All analyses are reported in the paper, in the main figures and tables, or in the Supplementary Material.

## Supplementary material

SUPPLEMENTARY FIGURE S1 Downregulated HLA class II molecules genes in relapsed CRC patients relative to disease-free. **(A)** HLA-DRA, **(B)** HLA-DRB1, **(C)** HLA-DMA, **(D)** HLA-DMB, **(E)** HLA-DQA1, **(F)** HLA-DPA1. The mean and 95% confidence interval values are represented by bold and light intermittent lines, respectively. Statistical significance was determined by Student’s t-test.

SUPPLEMENTARY FIGURE S2 PVR expression profile and disease-free survival according to MSI status. **(A)** Log2 PVR expression in MSI-high patients relative to MSI-low patients. The mean and 95% confidence interval values are represented by bold and light intermittent lines, respectively. Statistical significance was determined by Student’s t-test. **(B)** Free survival curves for MSI-low patients (blue) and MSI-high patients (red). The median gene expression value was used as the cut-off to stratify patients into the two cohorts, high and low. Hazard ratio (HR) and p-value were calculated using the log-rank test. The shaded area around each curve represents 95% confidence interval.

SUPPLEMENTARY FIGURE S3 Kaplan–Meier overall survival curve for patients with low PVR expression (blue) and patients with high PVR expression (red). The median gene expression value was used as the cut-off to stratify patients into the two cohorts, high and low. Hazard ratio (HR) and p-value were calculated using the log-rank test.

SUPPLEMENTARY FIGURE S4 Kaplan–Meier free survival curve for patients with low PVR expression (black) and patients with high PVR expression (red) in the validation dataset, GSE39582. The number at risk of relapse is shown under the curves. The median gene expression value was used as the cut-off to stratify patients into the two cohorts. Hazard ratio (HR) and p-value were calculated using the log-rank test. The figure was generated using KM plotter [36].

SUPPLEMENTARY TABLE S1 Clinical characteristics analysis.

SUPPLEMENTARY TABLE S2 Expression of various genes in relapse-free and relapsed CRC patients.

SUPPLEMENTARY TABLE S3 Genetic alteration.

SUPPLEMENTARY TABLE S4 The methylation status of various genes in relapse-free and relapsed CRC patients.

SUPPLEMENTARY TABLE S5 Most frequently mutated genes in the CRC cohort.

SUPPLEMENTARY TABLE S6 Clinical and genetic characteristics of patients with low *PVR* expression and patients with high *PVR* expression.
