# Supplementary figures and images for: Poliovirus receptor (PVR) expression as a predictor of relapse in colorectal cancer: bioinformatics and virtual screening
Source: Exp Biol Med (Maywood). 2026 Feb 13;251:10745. doi: 10.3389/ebm.2026.10745 (PMC12945842; doi:10.3389/ebm.2026.10745)

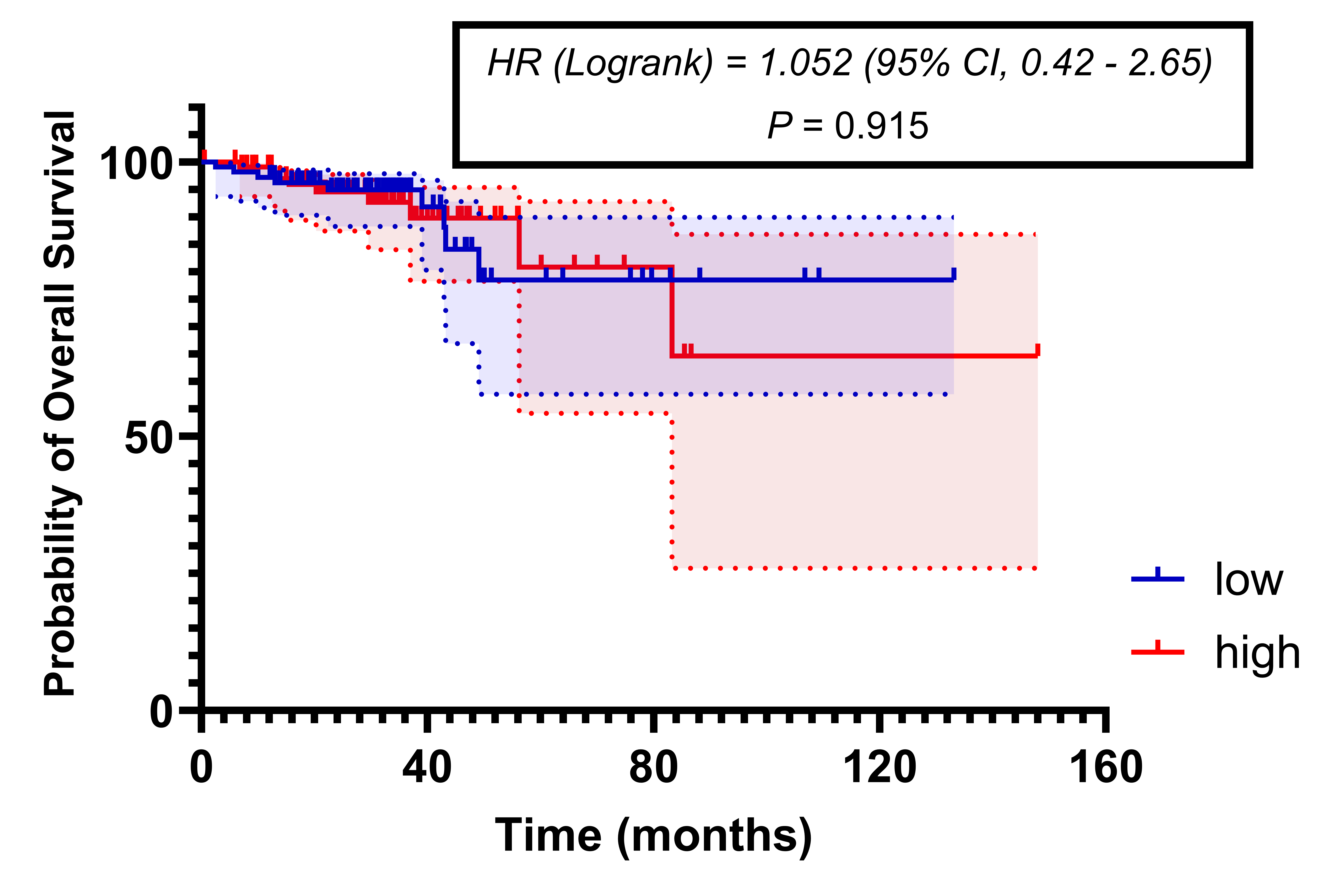

Supplement: Supplementary file 3 [file Image3.tif]

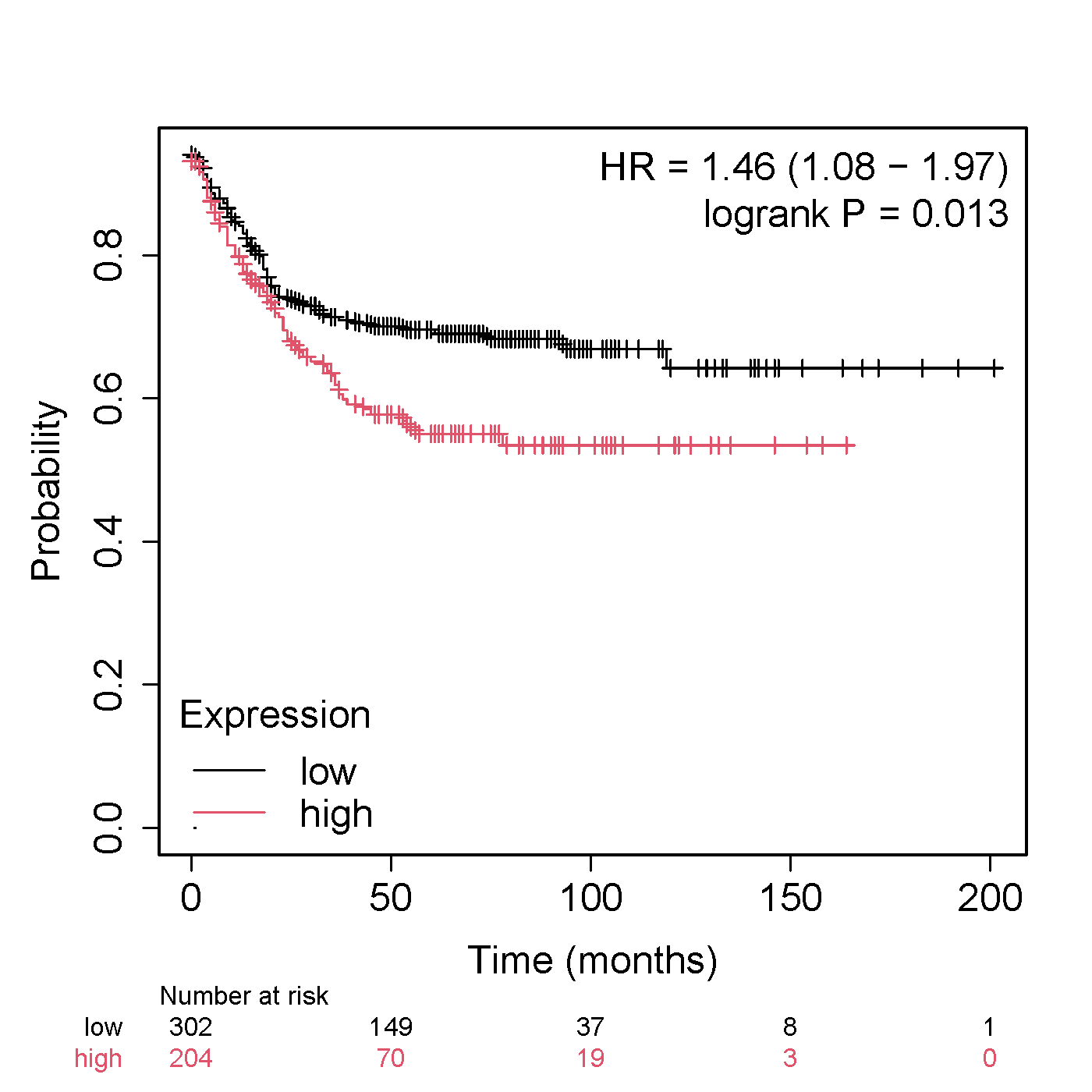

Supplement: Supplementary file 4 [file Image4.tif]

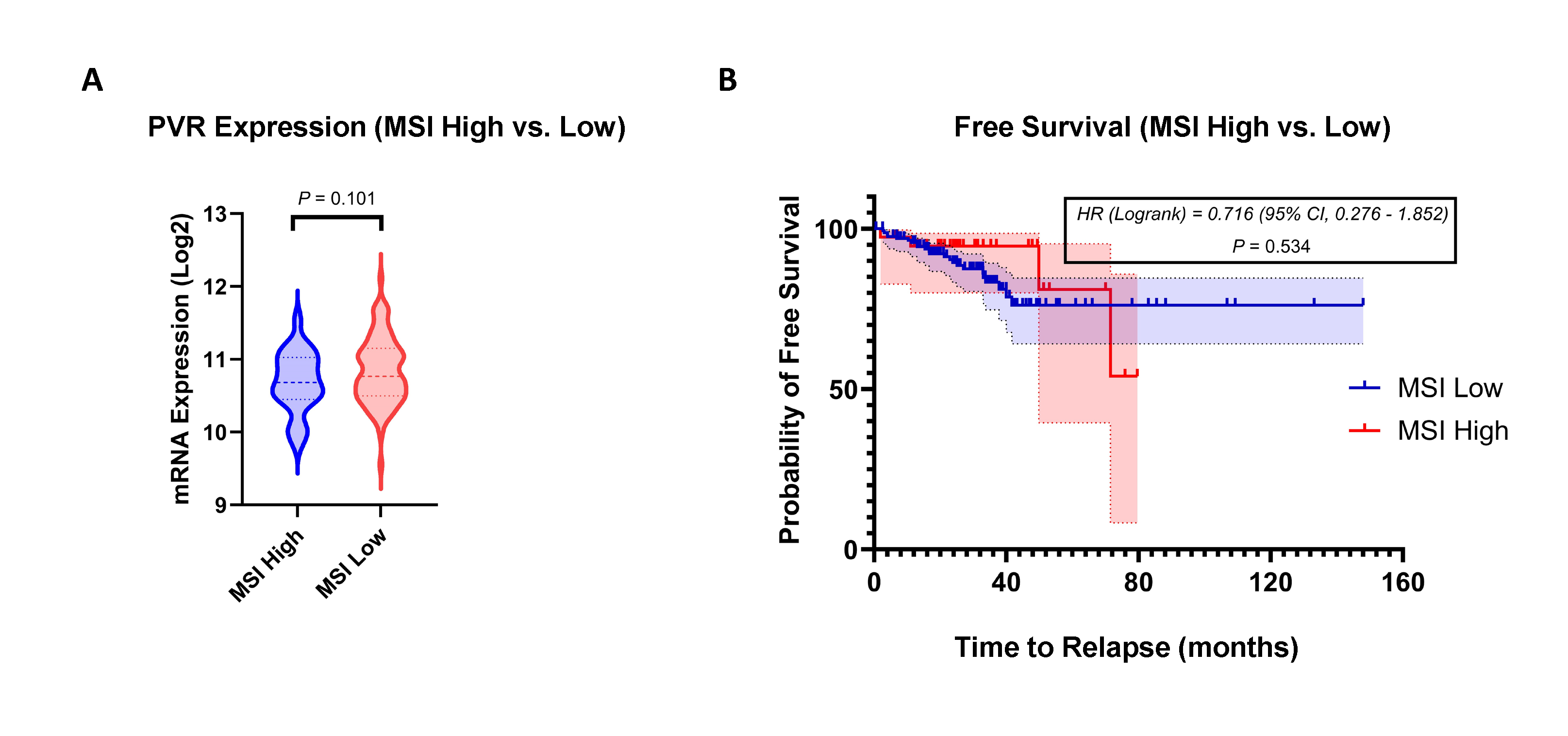

Supplement: Supplementary file 5 [file Image2.tif]

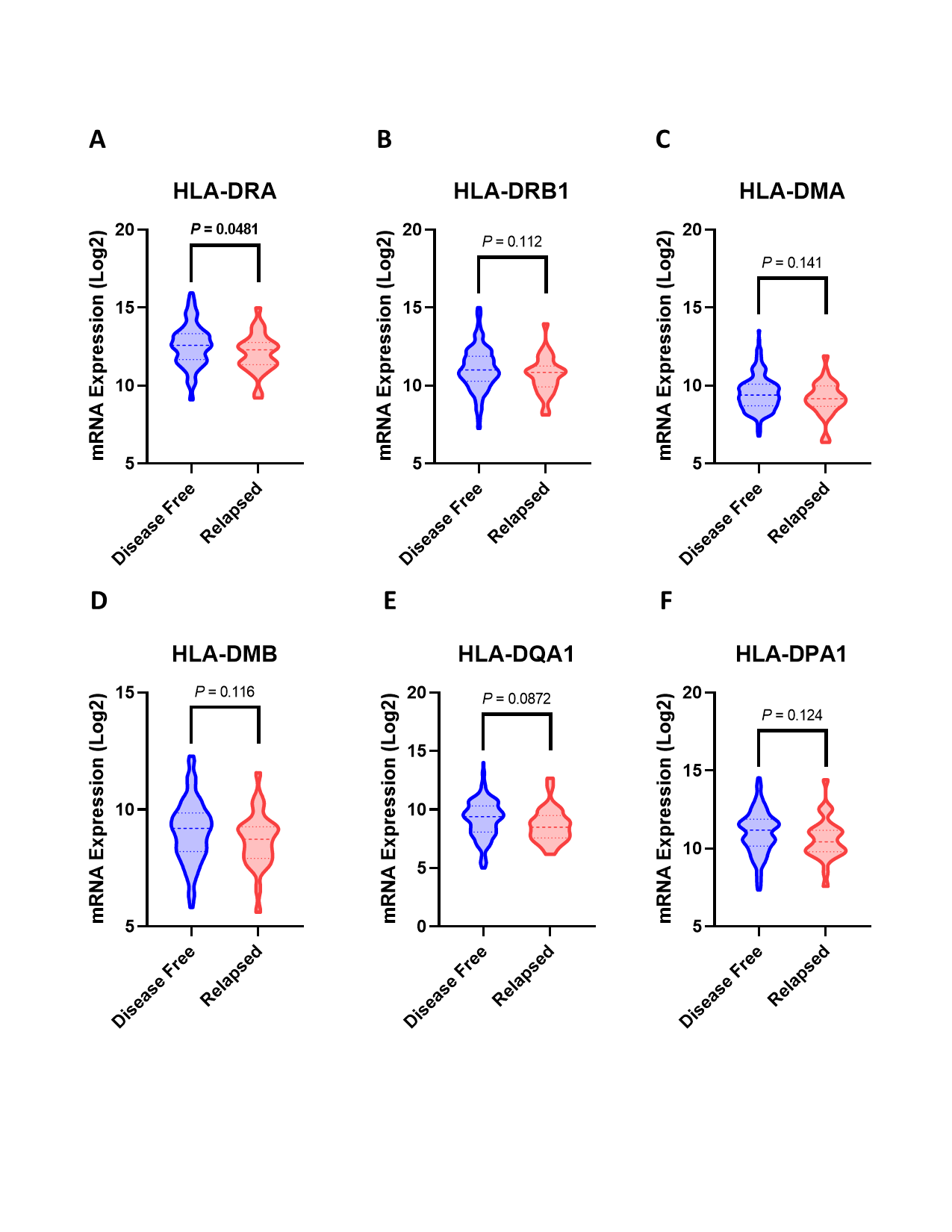

Supplement: Supplementary file 6 [file Image1.tif]
